# Colonisation and Diversification of the Zenaida Dove (*Zenaida aurita*) in the Antilles: Phylogeography, Contemporary Gene Flow and Morphological Divergence

**DOI:** 10.1371/journal.pone.0082189

**Published:** 2013-12-12

**Authors:** Karine Monceau, Frank Cézilly, Jérôme Moreau, Sébastien Motreuil, Rémi Wattier

**Affiliations:** 1 Université de Bourgogne, UMR CNRS 6282 Biogéosciences, Equipe Ecologie Evolutive, Dijon, France; 2 Institut Universitaire de France, France; University of Lausanne, Switzerland

## Abstract

Caribbean avifaunal biogeography has been mainly studied based on mitochondrial DNA. Here, we investigated both past and recent island differentiation and micro-evolutionary changes in the Zenaida Dove (*Zenaida aurita*) based on combined information from one mitochondrial (Cytochrome c Oxydase subunit I, COI) and 13 microsatellite markers and four morphological characters. This Caribbean endemic and abundant species has a large distribution, and two subspecies are supposed to occur: *Z. a. zenaida* in the Greater Antilles (GA) and *Z. a. aurita* in the Lesser Antilles (LA). Doves were sampled on two GA islands (Puerto Rico and the British Virgin Islands) and six LA islands (Saint Barthélemy, Guadeloupe, Les Saintes, Martinique, Saint Lucia and Barbados). Eleven COI haplotypes were observed that could be assembled in two distinct lineages, with six specific to GA, four to LA, the remaining one occurring in all islands. However, the level of divergence between those two lineages was too moderate to fully corroborate the existence of two subspecies. Colonisation of the studied islands appeared to be a recent process. However, both phenotypic and microsatellite data suggest that differentiation is already under way between all of them, partly associated with the existence of limited gene flow. No isolation by distance was observed. Differentiation for morphological traits was more pronounced than for neutral markers. These results suggest that despite recent colonisation, genetic drift and/or restricted gene flow are promoting differentiation for neutral markers. Variation in selective pressures between islands may explain the observed phenotypic differentiation.

## Introduction

Ever since Darwin [1], archipelagos have been regarded as natural laboratories for studying evolutionary patterns and processes of history of colonisation, population divergence and extinctions [2]–[5]. Islands are often characterized by both their level of isolation from mainland, implying restricted exchanges of individuals, and their small area limiting opportunities for population expansion. Basically, genetic variability in such systems is supposed to be low due to founder effect and genetic drift, especially when the size of the island is small [6], [7]. In archipelagos exhibiting different environments among islands, various selective pressures may promote genetic differentiation for morphological traits between units [8]–[10]. Alternatively, differentiation could be countered by the homogenising effect of migration between islands [11]. Indeed, previous studies of the patterns and processes of colonisation of islands and archipelagos have often revealed complex histories [2], [12], [13]. For instance, colonisations of islands are not necessarily concomitant with island geological age, can have multiple geographical origins, and can occur in discrete steps in time [14]. In addition, islands can sometimes be the source of colonisation of continental populations, although the reverse phenomenon is regarded as more common [15]. Finally, paleontological studies on island faunas have identified temporal variations in species richness as well as variable causes of extinctions [5].

Due to its geological history, the Caribbean archipelago, and especially the West Indies, is of particular interest for the study of species and population differentiation between islands and colonisation history [14], [16]–[18]. The West Indies consist of two forms of “true” island, as defined by Wallace [19]. The Greater Antilles (GA hereafter) are made up of continental fragments that remained more or less connected with North America until 49 Myr ago [20]. In contrast, the Lesser Antilles (LA hereafter) are mostly of volcanic or coral origin, with older islands originating in the late Eocene to Oligocene and younger ones having emerged in the late Miocene [21]. Barbados is known to be by far the youngest island, being only about 700 000 year old, compared to the remaining LA islands which are estimated to be 20–30 Myr older [22].

The Caribbean area is considered to be “a globally outstanding conservation priority ecoregion and biodiversity hotspot” for avifauna [23]. Caribbean bird populations offer a valuable opportunity to study how the Caribbean area was colonized and how genetic and morphological diversity can be structured both historically and through contemporary exchanges. To date, however, colonisation sources have been identified mostly from either North or South American mainland [14], [24]–[28]. In addition, the existing few studies have essentially relied on mtDNA paired with other markers such as morphometric characters [29], thus ignoring nuclear variation. Combining the two types of genetic markers plus morphometric characters offers, however, several advantages. First, because mtDNA markers are only maternally inherited while nuclear markers are biparentally inherited, their combination allows the detection of introgression in the case of an observed discrepancy between the results obtained with each type of markers (nucleo-cytoplasmic disequilibrium; see [14]). Second, a difference in mutation rate between markers might be informative of processes having taken place at different times in the evolutionary history of the taxa. For example, using mtDNA markers in combination with fast evolving markers such as microsatellite markers can provide information on contemporary gene flow [30], [31]. Third, morphological divergence may reflect local ecological constraints (e.g., competition, resource availability, predation pressure) which cannot be identified by neutral genetic markers (see [9] for example). Indeed, although the available information suggests that many Caribbean bird populations are isolated from each other [27], contemporary dispersal and gene flow between islands have not been assessed so far. Such studies may however provide a better understanding of micro-evolutionary processes [8].

The Zenaida Dove (*Zenaida aurita*) is widely distributed through the Caribbean area; its distribution ranging from the tip of the Yucatán Peninsula to the south of the Caribbean area [32]. Two plumage-coloration-based subspecies, *Z. aurita zenaida* (Bonaparte 1825) from *Z. aurita aurita* (Temminck 1810) have been described in GA and LA, respectively [32], [33]. Although the species is widespread over the Caribbean and considered to be abundant [34], illegal hunting and the recent introduction of two related competitive alien species, the Eurasian Collared Dove (*Streptopelia decaocto*) and the Ringed Turtle Dove (*Streptopelia risoria*) on some islands [35] may threaten Zenaida Dove populations. Thus, providing information about genetic structure, migration and potential local adaptation is of conservation importance. Previous studies have documented the behavioural ecology [36]–[39], morphological variation [40], demography [34], [41], [42], and phylogenetic relationships with other species belonging to the genus *Zenaida* [43], [44]. However, time and pathways of colonisation and diversification of the Zenaida Dove in the Antilles remain mostly unknown.

In the present study, eight island populations of the Zenaida Dove were sampled and data from one mtDNA marker, 13 microsatellite markers and four morphometric characters were combined and/or confronted in order to achieve three main objectives and to test associated hypothesis:

1. Identify historical processes of colonisation and expansion through the Caribbean area: Is the colonisation process associated with the age of islands? Are the two plumage-coloration-based subspecies supported by genetic and morphometric divergence?
2. Evaluate the contemporary diversity, gene flow and populations structuring within the area: To what extent genetic and phenotypic diversities are associated with island size? Does the distance between islands limit exchange and, therefore, promote genetic differentiation?
3. Evaluate the occurrence of selective processes through comparing morphological divergence with genetic divergence estimated from neutral genetic markers

## Materials and Methods

### Ethics Statement

All necessary permits were obtained for the described study (including bird catching/banding, blood sampling, and export/import of samples), which complied with all relevant regulations (details are given in Text S1).

### Study sites and field data collection

**Figure 1:**
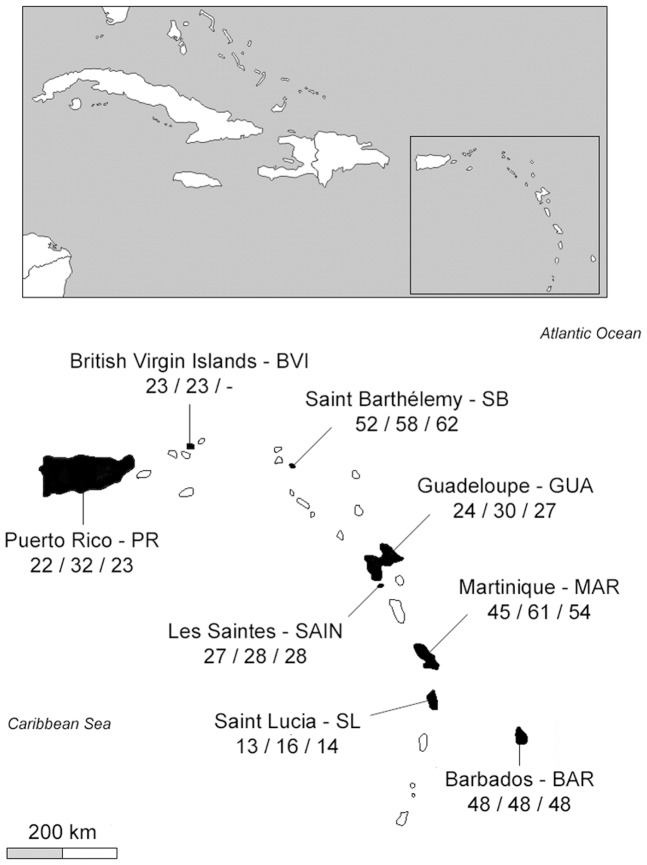
Map of Caribbean archipelago with sampled islands black. Site names with their acronym below are followed in parenthesis by sampling sizes for mtDNA, microsatellite and morphological analyses, respectively. PR and BVI are part of the Greater Antilles while all remaining islands are part of the Lesser Antilles.

Data were collected in two GA islands, Puerto Rico (PR) and the Guana Island in British Virgin Islands (BVI), and six LA islands: Saint Barthélemy (SB), Guadeloupe (GUA), Terre de Haut island in Les Saintes (SAIN), Martinique (MAR), Saint Lucia (SL) and Barbados (BAR) (Figure 1). The islands sizes ranged from 3.4 km^2^ (BVI) to 8870 km^2^ (PR). Geographical distances between islands were measured with Google Earth (http://earth.google.com) to the nearest kilometre, with distances ranging from 11 km (GUA-SAIN) to 836 km (PR-BAR) (Figure 1).

Zenaida Doves were caught using walk-in baited drop traps, single-catch closing net bird traps, or mist nets. Blood samples for DNA analyses were taken by puncture of the brachial vein and conserved in storage buffer (70% absolute ethanol, 30% TE pH 8). Morphological characters were obtained from all but one (BVI) islands: right tarsus length (digital calliper to the nearest 0.1 mm), tail and right wing chord (ruler to the nearest 1 mm). All measurements were taken twice (except in PR) to evaluate their repeatability. Birds were also weighed to the nearest 0.1 g (MS500, Pesola©, Switzerland). All birds were released at their capture point.

### Molecular methods

DNA was extracted according to a standard phenol-chloroform method, as described in Monceau et al. [45]. As sexual dimorphism is weak in *Z. aurita* [40], sex identification was based on intron size variation of the Chromo-Helicase-DNA binding protein (CHD1) gene, using primers described by Fridolfsson and Ellegren [46] which amplify two bands (CHD1-W = 450 pb and CHD1-Z = 750 pb) easily resolved on 2% agarose gels.

A total of 254 *Z. aurita* individuals (Figure 1) as well as seven *Z. galapagoensis*, a closely-related species [43] used here as an outgroup for the construction of a phylogenetic tree (see next section), were amplified for the 5′ part of the mtDNA Cytochrome c Oxidase subunit I (COI) using Bird-F1 and Vertebrate-R1 primers [47]. PCR products were Exo-SAP cleaned-up and sent to Macrogen (Macrogen Inc., Korea) for sequencing according to the Big Dye Sequencing protocol (Applied Biosystems 3730xl). Sequences were edited and aligned manually using Mega (v 5.05., [48]) leading to 627 bp long sequences with no indels or stop codons. Haplotypes were deposited in GenBank (Accession JN639022-32).

A total of 296 individuals (Figure 1) were genotyped at 13 microsatellite markers (Table S1) developed for *Z. aurita* [45]. PCR conditions for all three types of markers (sex, mtDNA and microsatellites) were as described in [45]. Microsatellite alleles were visualized on 6.5% acrylamide 41 cm long gels on a Licor 4200 L automated sequencer and scored independently by eye by two persons. Reference individuals were included for inter-gel calibration.

### Historical colonisation and demographic processes

In order to help visualizing the spatial distribution of mtDNA polymorphism and the extent of molecular divergence, a minimum spanning network was generated using Arlequin (v.3.5.1.3., [49]) based on pairwise absolute distances between haplotypes. Pairwise island differentiation was estimated by Φ_ST_ values, a F_ST_ analogue for mtDNA sequences, using Arlequin, in order to identify sets of islands that would be homogenous in haplotype frequencies and, therefore, be representative of historical phylogeographic units. Statistical significance was tested with 45000 permutations and 5% nominal level was adjusted for multiple comparisons with Benjamini-Yekutieli step-up procedure to control for false discovery rate (BY, [50]).

In order to search for molecular evidence supporting the existence of two subspecies, mean Kimura two parameters (K2p) genetic distance was calculated between haplotypes of the GA and LA islands lineages using Mega. In addition, a neighbour-joining (NJ) tree was produced using Mega based on K2p distances with 1000 bootstraps with Z. *galapagoensis* as outgroup. K2p and NJ were chosen as recommended by Tavares and Baker [51] for COI barcodes. Divergence time between GA and LA islands lineages was estimated using the mean number of differences between lineages and a 2% per Myr divergence rate, a value which is appropriate for the genus *Zenaida* [44].

For demographic history, two types of models were tested for mtDNA: 1) pure demographic expansion within islands and 2) spatial expansion associated with expansion-colonisation at the scale of a set of islands. First, following recommendations from Ramos-Onsins and Rozas [52], two different statistics were used to test for demographic expansion in each island presenting polymorphism. We first used Fu's Fs statistic [53] to test for an excess of low-frequency haplotypes in an expanding population compared to a stable one. We then relied on the more conservative square differences statistic (SSD, mismatch distribution, [54]) to compare the observed distribution of the number of nucleotide differences between haplotype pairs to the distribution expected under the null hypothesis of sudden expansion (unimodal distribution, [55]; see however [56] about the limited power such analysis might have). Second, the SSD test was also used to specifically test for an instantaneous spatial expansion e.g., an association between demographic expansion and instantaneous expansion-colonisation in a set of islands [57], [58]. For this analysis, islands not differing in haplotype frequencies were grouped, resulting in three different groups: PR + BVI (GA), SB-GUA + SAIN + MAR + SL (LA to the exception of BAR), and BAR (see results). Where relevant, expansion time in mutational units (τ) was obtained with 90% confidence interval by parametric bootstrapping (10000 replicates). All analyses were computed with arlequin. Time in years since expansion (t) was estimated as t = (τ/2u) * g, with *u* being the haplotype mutation rate (fixed at 2%, see above) and *g* being the generation time of the species. Generation time was calculated as *g* = µ+[s/(1−*s*)], where µ is the average age at first breeding and *s* is the adult survival rate [58]. Considering a minimal survival rate of 0.77 for adult reproductive females [42], and one-year old as a rough estimation of the age at which 50% of individuals have been recruited in the breeding segment of the population [34], [41] the generation time was estimated to be 4.35 years. Both statistics and their statistical significance were computed with Arlequin.

### Contemporary diversity, gene flow and population structuring

#### Based on microsatellite markers

Measures of genetic diversity, including the number of alleles (Na), the allelic richness (Ar) calculated by rarefaction to the smallest sampling size [59], and expected heterozygosity (He), were all computed with Fstat (v. 2.9.3.2., [60]). Between-island differences for Ar and He were tested with ANOVAs after checking for normality and variance homogeneity. The relationship between genetic diversity (mean Ar) and island size was tested using a Spearman's rank correlation coefficient [10], [61]. For each island, Hardy-Weinberg equilibrium (HWE) was tested for each microsatellite locus and for the complete set of loci, and linkage disequilibrium (LD) was tested for each pair of microsatellite loci, with statistical significance tested with 104000 and 12480 permutations, respectively, using Fstat. Nominal significance level (5%) was adjusted with BY's correction. Loci with excess of homozygous individuals were tested for the presence of null alleles using Micro-Checker [62].

As some loci in some islands were presenting null alleles (see results) island pairwise F_ST_ values were first generated with FreeNA [63], thus allowing the computation of F_ST_ values accounting or not for null alleles assorted with their 95% confidence intervals (95% CI). As all 95% CI overlapped, uncorrected values (as estimated from Weir and Cockerham [64] in Fstat) were used in further analyses. Second, following Raymond and Rousset [65], island pairwise differentiation was also tested by a contingency test of allele frequency heterogeneity using Arlequin. Contingency tests have been shown to be more powerful in detecting differentiation than F_ST_ estimators in the case of microsatellite data when sampling is unbalanced [66] and/or when differentiation level is low [67], as is the case in the present study.

To test if the distance between islands could be responsible for population differentiation, we tested isolation by distance (IBD) using a Mantel test with 20000 permutations between F_ST_/(1−F_ST_) and ln(geographic distance in km) using Fstat. Population structure was also analyzed using individual-based Bayesian clustering method implemented in Structure (v 2.2.3., [68]). No information about sample location was used to run the simulations using the admixture and correlated allele frequencies models to account for possible similar allele frequencies between populations [68]. Five independent simulations for a given *K* number of clusters were performed, from *K* = 1 to *K* = 8, each one with burn-in of 10^5^ iterations and a run length 10^5^ iterations following the burn-in. We then obtained a log-likelihood of the posterior probability of the data for a given *K* (ln Pr_(X|K)_). The posterior probabilities of *K* were computed with the *ad hoc* statistic based on Bayes' rule [68]. Following the recommendations of Waples and Gagiotti [67], we used this statistic rather than Evanno's test [69], as the observed level of differentiation was moderate (see results).

To identify dispersal pathway at the individual level, we evaluated the proportion of first generation (F_0_) migrants using Geneclass 2 [70], [71], and identified the most likely geographic origin of each identified F_0_ migrant using the Monte Carlo resampling method [72]. Since our sampling scheme did not cover all potential source populations, we relied on L_origin_, defined as the likelihood of extracting one individual with a given genotype from the population in which it was caught, for a given allele frequency distribution [72]. This likelihood was computed according to Paetkau et al. [72] resampling algorithm for 100 000 simulations and an alpha level of 1%.

Evidence for the reduction of population size (i.e., bottlenecks) occurring within islands across tens to hundreds of years was assessed through comparing the number of loci showing heterozygosity excess with the number of those showing heterozygosity deficit using Wilcoxon signed-rank tests [73]. Both estimates are expected to be identical for a neutral locus in a population at mutation-drift equilibrium. We used the two-phase model (TPM) provided by Bottleneck [74], [75], as it appears to be better suited to microsatellite markers [75], with 95% of single step mutation and 5% of multi-step mutation, and variance among multi-step of 12 and 10000 replications.

#### Based on morphometric characters

The repeatability of measurements (R) was first checked (except for PR for which we only had one measurement per individual) using linear mixed-effects model-based repeatability estimates (LMM), with restricted maximum likelihood for estimating unbiased variance components following Nakagawa and Schielzeth [76]. Significant variances in the random effects were tested with likelihood ratio tests, and R was given with its 95% confidence interval for each population. To test for overall morphological differentiation between islands, we first examined the influence of island, sex and their interaction on each morphological character using ANOVAs. Tukey Honestly Significant Difference tests (Tukey HSD tests) were used to identify significant differences between groups. Sexual dimorphism was compared between islands according to the method proposed by Santiago-Alarcon and Parker [77], using multiple pairwise comparison tests with BY's correction.

Population structure based on morphological characters was analysed using (*i*) model-based clustering and (*ii*) pairwise phenotypic index of differentiation between islands P_ST_ (an analogous to Q_ST_). Model-based clustering for Gaussian mixture models by expectation-maximisation algorithm was computed for *G* clusters from 1 to 7, based on the four traits. The best model was defined as having the best Bayesian Information Criterion (BIC) score. Then, P_ST_ based on both the first and the second axes of the Principal Component Analysis (PCA) including the four measurements was used to quantify the amount of between-population phenotypic variance in quantitative traits [78]. Briefly, the PCA was performed using a singular value decomposition of the centred and scaled (standardized) data matrix [79], and reversing the rotated matrix of principal component scores. We retained the first and the second axis of the PCA (thereafter noted PC1 and PC2) as they had both an eigenvalue above one and accounted for 48.28% and 26.14% of the overall variance in morphology, respectively. According to the factor loadings, PC1 reflects an overall measure of body size (tarsus length  = 0.58, tail length  = 0.43, wing chord  = 0.45 and body weight  = 0.52) whereas PC2 (tarsus length  = −0.27, tail length  = −0.68, wing chord  = 0.58 and body weight  = 0.35) may possibly reflect manoeuvrability and/or agility and escape performance (see [80], [81]). Following Wojcieszek and Simmons [82], P_ST_ values between pairs of island populations were then computed independently for PC1 and PC2 using ANOVAs, and the statistical significance of each pairwise differentiation was assessed using *F*-ratio tests associated with BY's correction [83]. In order to test for an effect of geographical distance on morphological differentiation between islands, a Mantel test was performed between P_ST_ and geographical distance using Fstat (20000 permutations).

### Morphological vs. genetic variation: do selective processes among island occur?

The evolution of microsatellite markers is generally considered to be driven by neutral (drift and gene flow) rather than selective processes [84]. Similarly, phenotypic variation can also be driven by none-selective processes [85]. If both assumptions hold true, then both genetic and morphological differentiation between islands can be positively correlated [8], [9], [86]. Occurrence of such a correlation (between P_ST_ distances and F_ST_ values) was tested in a Mantel test with untransformed matrices using Fstat (20000 permutations). Under directional selection, morphological differentiation will be larger than that observed at neutral markers [86]. Following Brommer [87] recommendations, we also provided 95% confidence intervals (95% CI) for P_ST_ (both PC1 and PC2) and F_ST_ based on 1000 bootstrap iterations. Non overlapping 95% CI values and P_ST_>F_ST_ are expected if directional selection is acting on morphological characters in addition to drift and restricted gene flow [82], [87].

Statistical analyses were performed using the R software (v. 2.12.1, [88]), implemented with the packages: *mclust* (model-based cluster analysis for Gaussian mixture models), *mutoss* (BY's corrections), *rptR* (repeatability estimation, [76]), and *bootstrap* (95% confidence interval bootstrapping).

## Results

### Historical colonisation and demographic processes

**Figure 2:**
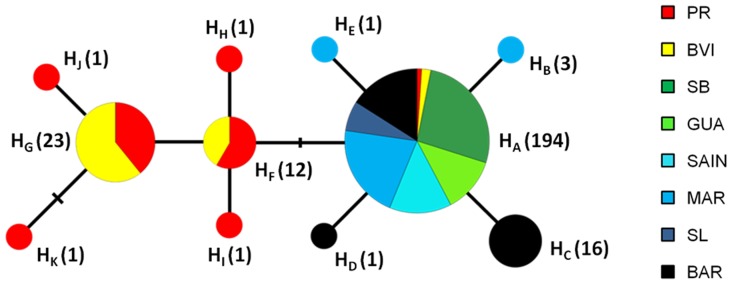
Minimum spanning network of 11 haplotypes (H_A_–H_K_) for 627 bp mtDNA COI sequences. Each circle represent a haplotype and the circle area is roughly proportional to the number of individuals sharing this haplotype (number of individuals harbouring a given haplotype is written in parenthesis, right to the haplotype name. Segments linking haplotypes are proportional to nucleotidic differences between haplotypes (one segment corresponding to one nucleotidic difference). Colours and its surface within a circle refer to the eight sampled islands and the relative abundance of a given haplotype, respectively. See Figure 1 for island acronyms.

The 627 bp long COI sequences revealed 11 haplotypes (H_A_–H_K_) associated with polymorphism at 10 single nucleotides, all being synonymous except one associated with the definition of haplotype H_K_, observed in only one individual (Figure 2, Table S2). The 11 haplotypes showed a contrasted pattern of geographic distribution. The number of haplotypes differed markedly between islands, ranging from one single haplotype up to seven in the most diverse GA island (PR). H_A_ was the most frequent haplotype in LA ranging from 65% (BAR) to 100% (SB, GUA, SAIN, SL), but was rare in GA (PR: 9%; BVI: 17%). Other haplotypes were either specific to GA (six haplotypes, H_F_–H_K_) or LA (four haplotypes, H_B_–H_E_, Figure 2).

A maximum of six nucleotide differences was observed between the most divergent haplotypes (Figure 2). The mean K2p genetic distances between GA haplotypes (H_F_–H_K_) vs. LA haplotypes (H_A_–H_E_) was 0.0067 (95% CI: 0.0052–0.0082). In comparison, K2p genetic distance between *Z. aurita* and Z. *galapagoensi*, its closest relative, was 0.0387 (95% CI: 0.0397–0.04243). The Neighbour-joining tree supported with a 100% bootstrap value these two haplotypes groups (H_A_–H_E_ vs. H_F_–H_K_) as being two phylogenetic lineages (Figure S1).

Pairwise island Φ_ST_ values revealed no differentiation between the two GA islands (PR and BVI), whereas these two islands were differentiated from all Lesser Antillean islands (0.6564<Φ_ST_<0.8112, Table 1). Among LA islands, only Barbados was differentiated from all other islands (0.1894<Φ_ST_<0.3100, Table 1).

**Table 1:** Pairwise island mtDNA θ_ST_ values (above diagonal) and microsatellite F_ST_ values (below diagonal) of Zenaida Doves (*Zenaida aurita*) for eight Caribbean islands.

	PR	BVI	SB	GUA	SAIN	MAR	SL	BAR
PR	-	−0.0136 ^NS^	**0.8112** ***	**0.7226** ***	**0.7363** ***	**0.7577** ***	**0.6564** ***	**0.7088** ***
BVI	0.0250 **	-	**0.8389** ***	**0.7612** ***	**0.7730** ***	**0.7820** ***	**0.7042** ***	**0.7262** ***
SB	0.0526 ***	0.0357 ***	-	0.0000 ^NS^	0.0000 ^NS^	0.0397 ^NS^	0.0000 ^NS^	**0.3100** ***
GUA	0.0706 ***	0.0541 ***	0.0554 ***	-	0.0000 ^NS^	0.0117 ^NS^	0.0000 ^NS^	**0.2331** ***
SAIN	0.0849 ***	0.0558 ***	0.0658 ***	0.0768 ***	-	0.0159 ^NS^	0.0000 ^NS^	**0.2428** ***
MAR	0.0649 ***	0.0419 ***	0.0477 ***	0.0524 ***	0.0631 ***	-	−0.0131 ^NS^	**0.2436** ***
SL	0.0826 ***	0.0512 ***	0.0391 ***	0.0653 ***	0.0789 ***	0.0202 ***	-	**0.1894** *
BAR	0.0974 ***	0.0608 ***	0.0527 ***	0.0705 ***	0.0870 ***	0.0553 ***	0.0563 ***	-

*: 5% nominal level, **: 1% nominal level and ***: 0.1% nominal level). See Figure 1 for island acronyms. Significant level after BY's correction in bold (NS: not significant,

Demographic scenario tests were performed within the four islands presenting polymorphism (PR, BVI, MAR and BAR). Results from demographic tests clearly supported a within-island demographic expansion for MAR (Fu's Fs = −1.694, *P* = 0.038 and SSD  = 0.00834, *P* = 0.401). For PR, the null hypothesis of an expansion could not be rejected using SSD test (SSD  = 0.0068, *P* = 0.550), while Fu's Fs test was slightly less conclusive at rejecting the null hypothesis of demographic stability (Fs = −2.043, *P* = 0.093). For the two remaining islands, results were in agreement with demographic stability (BVI: Fs = 1.598, *P* = 0.871 and SSD  = 0.4357, *P*<0.001, and BAR: Fs = 0.270, *P* = 0.565 and SSD  = 0.0256, *P* = 0.042). The SSD tests for GA and LA (minus BAR) were in agreement with a spatial expansion (GA: SSD  = 0.00834, *P* = 0.401 and LA: SSD  = 0.0002, *P* = 0.273). The average time since spatial expansion was estimated to be 54 000 years ago (90% CI: 52–544) and 18 000 years ago (90% CI: 0–109) for the GA and LA minus BAR, respectively.

### Contemporary diversity, gene flow and population structuring

#### Based on microsatellite markers

Contrasting with mtDNA (see previous paragraph), microsatellite diversity was observed in each island. The mean number of alleles (Na) per island ranged from 6.23 (SL) to 10.08 (PR) (Table S1). Microsatellite allelic richness (Ar) differed between islands (ANOVA: *F*_7,96_ = 3.64, *P*<0.01), but not for expected heterozygosity (He, *F*_7,96_ = 1.25, *P* = 0.28, Table S1). No relationship was found between Ar and island size (Spearman rank-correlation coefficient, r_s_ = 0.09, *P* = 0.84, *n* = 8). No evidence for LD between pairs of loci was found in any island. Three populations, PR, BVI and SB, showed multilocus deviation from HWE, caused by only two or three loci, depending of the island (Table S1) that were all prone to null alleles, as identified with Micro-checker.

All island pairwise F_ST_ values were significantly different from zero (0.0202–0.0974, Table 1), 18 out of 21 being higher than 0.05, thus indicating a moderate level of differentiation. The pattern of differentiation was confirmed by the exact test of differentiation, all comparisons being highly significant (data not shown). No isolation by distance (IBD) was present as no significant correlation was detected between geographical distance and genetic differentiation (Mantel test, R^2^ = 0.07, *P* = 0.18).

Based on Structure simulations, the sampled individuals could be grouped in seven clusters (*P*_(K = 7)_ = 1, Figure S2). MAR and SL then appeared to form a homogenous group whereas each other island represented a single genetic unit. Eight individuals (three females and five males) were identified as F_0_ migrants (2.70%, Table 2). Interestingly, F_0_ migrants were not only detected between neighbouring islands like BVI and PR (distant from about 95 km and unified during glaciations due to low sea level), but also from SB to PR (291 km), from SB to MAR and reciprocally (377 km), from BVI to SAIN (424 km), from PR to MAR, (597 km), and even from BVI to BAR (772 km, Table 2).

**Table 2:** Detection of first generation (F_0_) migrants assessed by Geneclass 2 of Zenaida Dove (*Zenaida aurita*) as a percentage of individuals (absolute number in parenthesis) assigned to a putative island of origin given the island were individuals were sampled in.

		Assigned to (%)							
Sampled in	N	PR	BVI	SB	GUA	SAIN	MAR	SL	BAR
PR	32	**87.50**	**9.375 (1-H_F_, 2-H_G_)**	**3.125 (1-H_I_)**	0.0	0.0	0.0	0.0	0.0
BVI	23	0.0	**100.0**	0.0	0.0	0.0	0.0	0.0	0.0
SB	58	0.0	0.0	**98.28**	0.0	0.0	**1.72 (1-H_A_)**	0.0	0.0
GUA	30	0.0	0.0	0.0	**100.0**	0.0	0.0	0.0	0.0
SAIN	28	0.0	**3.57 (1-H_A_)**	0.0	0.0	**96.43**	0.0	0.0	0.0
MAR	61	0.0	0.0	**1.64 (1-H_A_)**	0.0	0.0	**98.36**	0.0	0.0
SL	16	0.0	0.0	0.0	0.0	0.0	0.0	100.0	0.0
BAR	48	0.0	**2.08 (1-H_A_)**	0.0	0.0	0.0	0.0	0.0	**97.92**

=  sampling size. See Figure 1 for island acronyms. N

Finally, no bottleneck was detected in any island (all Wilcoxon signed-rank tests *P*-values >0.07). These results provide support for the absence of founder effects or population expansion occurring across tens to hundreds of years in any of the eight islands.

#### Based on morphometric characters

**Figure 3:**
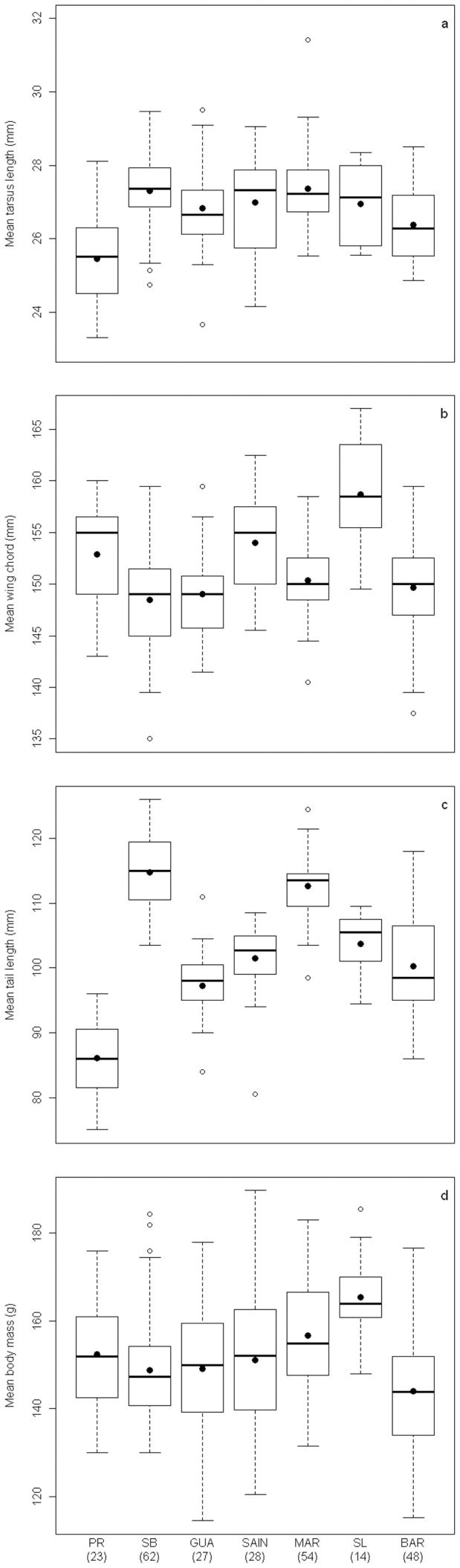
Morphological differences between islands in adult Zenaida Doves. a) tarsus length, b) wing chord, c) tail length and d) body mass. Boxes, plain line, black points, dash lines and open circles represent 50% of all values, medians, means, 1.5 x interquartile ranges and extreme values respectively.

Overall morphometric measurement error (ME) was low (tarsus length: 3.00%, wing chord: 2.80% and tail length: 2.20%), R was high (R>0.935) and the majority of 95% CI overlapped, thus justifying the use of the means of repeated measurements for subsequent analyses (Table S3). Each morphological character varied significantly between islands but no general trend appeared (Table 3 and Figure 3). Males were always larger or heavier than females (Table 3, Tukey HSD tests, all *P*<0.0001), and sexual dimorphism differed between islands only for wing chord and body mass (Table 3), with no general trend (Table 4).

**Table 3:** Results from ANOVAs testing for the effect of island, sex and their interaction on four morphological characters for *Zenaida aurita*.

Morphological character	Effect	*F*	dfs	*P*
Tarsus length	**Island**	**14.62**	**5, 242**	**<0.0001**
	**Sex**	**60.44**	**1, 242**	**<0.0001**
	Island x Sex	0.45	5, 242	0.84
Wing chord	**Island**	**22.50**	**5, 242**	**<0.0001**
	**Sex**	**128.44**	**1, 242**	**<0.0001**
	**Island x Sex**	**3.45**	**5, 242**	**<0.01**
Tail length	**Island**	**119.48**	**5, 242**	**<0.0001**
	**Sex**	**26.66**	**1, 242**	**<0.0001**
	Island x Sex	1.52	5, 242	0.17
Body mass	**Island**	**8.34**	**5, 242**	**<0.0001**
	**Sex**	**39.33**	**1, 242**	**<0.0001**
	**Island x Sex**	**2.52**	**5, 242**	**0.02**

Significant results are in bold.

**Table 4:** *P*-values (after BY's correction) associated with multiple pairwise comparison tests for sexual dimorphism analyses for wing chord (above diagonal) and body mass (below diagonal).

	PR	SB	GUA	SAIN	MAR	SL	BAR
PR	-	**0.01**	0.89	**<0.01**	**<0.01**	**<0.0001**	1.00
SB	1.00	-	0.09	1.00	**<0.0001**	**<0.0001**	**<0.01**
GUA	**<0.001**	**<0.01**	-	**<0.01**	**<0.0001**	**<0.0001**	0.46
SAIN	**<0.0001**	**<0.0001**	0.19	-	**<0.0001**	**<0.0001**	**<0.01**
MAR	0.11	**0.01**	**<0.0001**	<0.0001	-	**<0.0001**	**<0.01**
SL	1.00	1.00	**<0.0001**	**<0.0001**	**0.03**	-	**<0.0001**
BAR	0.52	0.08	**<0.0001**	**<0.0001**	1.00	0.19	-

Figure 1 for island acronyms. See

According to model-based clustering analysis, the best model (ellipsoidal covariance matrix with equal variance) was defined for three clusters (BIC  = −6299.393, next best model BIC  = −6319.019). At least 75% of the doves within each island were assigned to one specific group: PR constituted the first group, SB and MAR the second one, and GUA, SAIN, SL and BAR the last one (Table 5). According to overall body size (PC1), P_ST_ values of Zenaida Doves from SB, SAIN, MAR and SL differed from those from PR and BAR (Table 6). Although GUA was more closely related to the group formed by PR and BAR, birds from this island did not differ from those banded in SAIN. Most Zenaida Dove populations differed from each other based on agility and escape performance (PC2) except PR and SL, GUA and SAIN, and GUA and BAR (Table 6). Geographical distance was not correlated to morphological differentiation based on P_ST_ (Mantel tests: P_ST_-PC1: R^2^ = 0.007, *P* = 0.73 and P_ST_-PC2: R^2^ = 0.002, *P* = 0.85).

**Table 5:** Distribution of the 256 Zenaida Doves in the three clusters defined by the model-based cluster analysis of morphological data.

	G1	G2	G3	Total
PR	**18**	0	5	23
SB	0	**62**	0	62
GUA	0	0	**27**	27
SAIN	1	1	**26**	28
MAR	0	**50**	4	54
SL	0	0	**14**	14
BAR	1	11	**36**	48
Total	20	124	112	256

Figure 1 for island acronyms. See

**Table 6:** Pairwise island P_ST_ values for morphological data (PC1 and PC2 above and below the diagonal respectively) Zenaida Doves (*Zenaida aurita*) for seven Caribbean islands.

	PR	SB	GUA	SAIN	MAR	SL	BAR
PR	-	0.9382***	0.6132	0.8628***	0.9636***	0.9452***	0.5157
SB	0.9955***	-	0.8306**	0.0901	0.6608	0.7716	0.9272***
GUA	0.9837***	0.9845***	-	0.6340	0.9162***	0.8865***	0.2257
SAIN	0.9611***	0.9869***	0.6893	-	0.6271	0.6886	0.8283**
MAR	0.9943***	0.9429***	0.9654***	0.9744***	-	0.5423	0.9607***
SL	0.7636	0.9909***	0.9623***	0.8720***	0.9882***	-	0.9391***
BAR	0.9832***	0.9819***	0.3593	0.8154**	0.9440***	0.9545***	-

*: 5% nominal level, **: 1% nominal level and ***: 0.1% nominal level). See Figure 1 for island acronyms. Significant level after BY's correction in bold (NS: not significant,

### Morphological vs. genetic variation: do selective processes among island occur?

No significant correlation across islands was observed between F_ST_ and P_ST_ (Mantel tests: P_ST_-PC1: R^2^ = 0.008, *P* = 0.70 and P_ST_-PC2: R^2^ = 0.105, *P* = 0.15). P_ST_ values were always higher than F_ST_ ones (mean value assorted with 95%CI; F_ST_: 0.0638 [0.0627–0.0648]; P_ST_ - PC1: 0.7307 [0.7164–0.7450]; P_ST_ - PC2: 0.9088 [0.9002–0.9174]) with no overlap between F_ST_ and P_ST_ suggesting that directional selection is acting on morphological characters in addition to drift and restricted gene flow.

## Discussion

Combining and/or confronting results from the three data sets, i.e., mtDNA, microsatellites, and morphometrics, allowed the reconstruction of a micro-evolutionary scenario for both colonisation and currently observed differentiation at the scale of the eight studied islands. MtDNA showed a shallow divergence, but was geographically structured in three units: the GA, the LA (without BAR), and BAR. Both molecular and morphological markers confirmed this subdivision, and also revealed a significant level of differentiation between islands.

### Timing of divergence and historical spatial dynamic

The Antilles, and especially LA, colonisation history has been extensively studied for land birds with mtDNA markers, but almost exclusively in passerine species [14], [15], [24]–[26], [28], [29], [89]–[91]. These studies showed that there is no unique pattern of colonisation for the West Indies, but instead a huge diversity of patterns, with colonising times being scattered across taxa, with possible multiple colonisations both in space and time and even some islands being the source of continental populations.

In a recent study based on a multiple-gene Bayesian phylogeny (including COI), Johnson and Weckstein [44] estimated the divergence time to the most recent common ancestor between *Z. aurita* and a sister group (including *Z. macroura*, *Z. graysoni*, *Z. auriculata* and *Z. galapagoensis*) to be a maximum of 2 Myr ago. This time is also likely to represent the time of first colonisation of *Z. aurita* into the Caribbean, but no definitive evidence is currently placing the root of the Zenaida genus being outside the Caribbean. Data from the same study allow estimating the COI molecular clock at 2% divergence Myr^−1^ for the genus *Zenaida* [44]. Therefore, the mean allopatric divergence between the GA and the LA lineages within *Z. aurita* in our present study being 0.53% (3.3 mutations on average out of 627 bp long sequence), the associated divergence time may not exceed 250 000 years. It indicates that GA and LA Zenaida Doves lineages have persisted as two evolutionary isolated units through several Pleistocene glaciation cycles. We cannot directly state whether this allopatric divergence is associated with colonisation from GA to LA or the reverse. On one hand, the presence of continental population of Zenaida Doves in Yucatán is in favour of the first scenario but several species of birds have also colonized the Yucatán from the West Indies [15]. Additional sampling in the north-western part of the Caribbean up to Yucatán would help answering this question.

In both GA and LA, historical demographic analyses revealed recent instantaneous demographic expansion mediated by a spatial expansion between islands dating back ca. 54 000 and 18 000 years ago, respectively (although large CI should be kept in mind). PR and MAR are likely to be the source populations as they both harbour rare haplotypes and are also characterized by within-island demographic expansion. Therefore, two scenarii might explain the present day distribution of Zenaida Dove. First, PR and MAR might have been the first two islands to be colonized, with colonisation within each group of islands occurring more recently. Second, the whole archipelago might have been early colonized, followed by an extinction/re-colonisation event from PR and MAR (i.e., the taxon cycling hypothesis [26], [92]–[94]. Taxon cycling refers to sequential phases of expansions and contractions in species distributions associated with ecological and morphological shifts. However, testing this hypothesis would require fossil and paleoecological data [5] and the first hypothesis remains the most parsimonious one. It has been pointed out that extinction and (re-)colonisation dynamics might be driven by many factors including abiotic (e.g., climatic oscillations) and biotic (e.g., dispersal abilities, inter-specific competition and host parasite relationships) factors [95]. Indeed, many columbid species show high vagility and ability for long-distance flight [33]. First generation migrant analysis based on microsatellites suggests that long-distance dispersal might be possible for *Z. aurita*, as some individuals were captured more than 750 km away from their presumed natal island. However, such long-distance dispersal might not necessarily correspond to a single long distance flight, as the archipelago architecture offers many possibilities for stopovers. Veech et al. [96] showed that the related White-winged Dove (*Zenaida asiatica*) was able to colonise southern USA in a few decades, the extent of the expansion being strongly influenced by the availability of suitable habitats. The existence of rapid colonisation of an archipelago is known for other columbids like the Emerald Dove, *Chalcophaps indica*, in Vanuatu [97].

Barbados is presenting a peculiar situation and deserves specific attention. On the one hand, all three haplotypes present on the island (i.e., the widespread haplotype H_A_ and the two private haplotypes H_C_ and H_D_) belong to the LA lineage. Such a pattern is consistent with a recent colonisation of Barbados from other LA islands. Such a pattern has also been observed for the grassquit, *Tiaris bicolour*, and the common ground dove, *Columbina passerina* [89]. On the other hand, one of the two private haplotypes, H_C_ is present at a rather high frequency (33%). Indeed, Barbados was highly differentiated for haplotype frequencies from all other LA islands as measured by mtDNA Φ_ST_ values (all >0.65). This pattern would fit with the introduction of H_C_ associated with a strong founder effect which drastically modified initial frequency of H_C_ at the time of colonisation. Alternatively, H_C_ may correspond to a mutational event that occurred after the colonisation of BAR by Zenaida Doves, or might originate from more southern islands such as Saint Vincent and the Grenadines or Grenada.

To a certain extent, our results are compatible with the proposed taxonomic subdivision based on plumage coloration [32], [33], with one subspecies (*Z. aurita zenaida*) occurring in the GA, and another one (*Z. aurita aurita*) occurring in the LA. First, reciprocal monophyly and the observed fixed mutational difference are reflecting phylogenetic divergence [51], [98]. Second, the molecular divergence between these two lineages remained shallow (mean K2p distance 0.67%), well below the 2% threshold classically used in DNA barcoding to distinguish species, including birds [99], [100] and, in particular, four columbid genera [47]. However, it is also significantly lower than the 1% threshold often considered as representative of intra-specific polymorphism [99]. Third, the geographic distribution of each lineage mirrors the distribution previously ascribed to the two supposed subspecies, being mainly restricted to the GA (H_F_–H_E_) vs. the LA (H_A_–H_E_). The open sea stretch between GA and LA (the Anegada Gap), although relatively small, thus seems to be sufficient to promote the observed biogeographic divide between the two *Z. aurita* lineages. The Anegada Gap is indeed often recognized as a significant biogeographical divide in the West Indies [32] (but see [14] for a counter-example). However, the most abundant LA haplotype (H_A_) is also observed, although at low frequency, in GA. In addition, the six *Z. aurita* individuals harbouring haplotype Ha sampled in GA were associated with a nuclear background (microsatellite) “typical” of the GA area (not identified as F_0_ migrants), a pattern compatible with restricted gene flow involving introgression but also incomplete lineage sorting [14]. Finally, the cluster analysis of morphological variation offers some additional support as one cluster was almost exclusively represented by PR individuals characterized by smaller tarsus and tail length. Further sampling on other GA islands is required in order to better assess the extent of variation between islands. Our data support the recognition of two Molecular Operational Taxonomic Units (MOTUs). Determining true taxonomic status will require further examination, including patterns of mate choice. Future studies should also evaluate the extent of differentiation in plumage coloration between the different haplotypes using objective techniques of measurement such as reflectance spectrometry [101]. This might be both of evolutionary and conservation interest [102]. Even if *Z. aurita* is a recent species, species age is known to be a bad predictor of subspecies richness [103]. Taken together, these results are in contrast with the often more complex pattern observed for Antillean passerine birds for which concordance is often challenged (e.g., [14], [24], [25]). A shallow phylogeographic divergence has also been observed in two North-American *Zenaida* species [104], [105]. However, whereas no congruence with conventionally recognized subspecies was observed for the Mourning Dove, *Z. macroura* [104], the opposite was true for the White-winged Dove, *Z. asiatica*, in which genetic differentiation was associated with morphological differentiation [105].

### Contemporary gene flow, drift and selection

Each island population was found to be at HWE for microsatellites, and no bottleneck was detected in any population. No difference between islands was detected for expected heterozygosity (He) although a difference in microsatellite allelic richness (Ar) was significant. However, no relationship was found between Ar and island size, although Ar extreme values were observed for the smallest and largest islands, respectively (SAIN vs. PR: 5.80 vs. 8.10). Therefore, island size does not seem to be a good predictor of the effective population size. Caribbean islands of small or intermediate size might offer a variable surface of suitable habitat for the Zenaida Dove. A similar pattern has been observed in the Galápagos Dove across five islands, using a set of five microsatellite markers [106]. Unfortunately, no study of a Caribbean bird species using microsatellites is available in the literature for comparison.

Differentiation for microsatellite markers was observed between any pair of islands as measured by F_ST_ (most values >0.05). This pattern was largely in accordance with individual-based Bayesian clustering. Seven different clusters were recognized, with MAR and SL constituting one single unit, which is congruent with this pair of islands having the smallest F_ST_ value (0.0202). Two evolutionary forces could explain this pattern of differentiation: restricted gene flow and drift. However, linear distance alone was not sufficient to account for the observed levels of differentiation as no isolation by distance was detected. Other factors, such as prevailing wind [107] and/or accidental (stowaway) or active or passive transportation of birds on the numerous ships and sailing vessels that regularly cruise between Caribbean islands may also affect the observed levels of genetic differentiation between islands. Although columbids are often recognized as good flyers, potential dispersal associated with such capabilities might not be realized. For the plain pigeon, *Columba inornata*, Young and Allard [108] showed (based on mtDNA haplotype frequencies) that gene flow was reduced between the Dominican Republic and Puerto Rico which are only 127 km apart. On the contrary, gene flow was high and no significant differentiation was observed between five Galápagos Islands in the Galápagos Dove [106].

Five F_0_ migrants moved within either LA or GA (Table 3). Not surprisingly, they belonged to a frequent haplotype in these areas. Such migrants could be either short (e.g., PR-BVI) or long (e.g., SB-MAR ca. 400 km) distance migrants. Long distance migrants might benefit from regularly spaced islands within the archipelago as stopovers. One migrant from SB (LA) to PR (GA) was the unique representative of the H_i_ haplotype, thus offering little possibility of interpretation. The two last migrants were from BVI (GA) to either SAIN (LA) or MAR (LA), and were also surprisingly associated with a rare haplotype in GA (Ha). Explaining such a pattern would imply that the observed migrant is a descent of a LA female harbouring Ha haplotype which initially migrated to GA, being introgressed there by GA individuals. Shared ancestral polymorphism for the Ha haplotype might however be an alternative, more parsimonious, explanation.

Molecular divergence for mtDNA is only partially reflected in morphological differentiation as five islands SB, SAIN, GUA, SL and MAR are not differentiated for mtDNA (all islands harbour 91–100% of Ha haplotype), while being morphologically differentiated. A similar discrepancy between mtDNA divergence and morphological differentiation has been previously observed in the Adelaide's Warbler, *Setophaga adelaidae* between Puerto Rico, Barbuda, and St. Lucia [30]. Similarly, the Australian mainland and island subspecies of the Silver White-eye *Zosterops lateralis* were documented to share the same haplotypes while being morphologically differentiated [109].

Overall body size and body mass differed between sexes with males being larger or heavier than females, confirming earlier results [36], [37], [40]. Between islands variation in sexual dimorphism was observed (wing chord and body mass), with no general trend, contrary to what has been observed in *Z. galapagoensis* [77]. Each morphological character varied significantly between islands but geographical distance did not explain morphological divergence.

No significant relationship was observed between morphological variation and genetic differentiation for microsatellites between islands. Phenotypic differentiation (P_ST_) was always higher than genetic differentiation (F_ST_), suggestive of selection pressures acting on morphological characters [82], [87]. However, these results should be interpreted cautiously since P_ST_ reflects both the genetic and environmental contribution to phenotypic variation [110]–[112]. Thus, the most parsimonious hypothesis to explain the observed morphological differentiation, including island variation in sexual dimorphism, in Zenaida Doves is probably a mix of different ecological pressures coupled with drift and restricted gene flow as revealed by microsatellites. Such a pattern has been observed (using F_ST_-P_ST_ approach) for plumage colour variation in the pied flycatcher (*Ficedula hypoleuca*, [113]) and body mass in Finnish House Sparrow (*Passer domesticus*, [114]). It has also been documented in the Silvereye *Zosterops lateralis* [8], where restricted gene flow and drift alone are not sufficient to explain observed morphological differentiation. Morphological differentiation might be extremely rapid even in the presence of gene flow as exemplified by the Elepaio, *Chasiempis sandwhichensis*, a Hawaiian monarch flycatcher for which no microsatellite differentiation was observed while pronounced morphological divergence was present, associated with a steep climatic gradient [115]. The clustering of some islands according to the model-based cluster analysis, SB-MAR and GUA-SAIN-SL-BAR, may possibly reflect convergent ecological pressures, although ecological differences and similarities between and within the two groups are not obvious. A similar pattern has been however observed in the Berthelot's pipit, *Anthus berthelotii* [9], the Island Canary, *Serinus canaria* [116], the Vanuatu white-eye, *Zosterops flavifrons* [117].

### Conclusion

In this study, we have described the historical pattern of Zenaida Dove colonisation through the Caribbean area, including subspecies within the Antilles (*Z. aurita zenaida* occurring in GA, and *Z. aurita aurita* occurring in LA). We have also identified the current limited gene flow between islands, and revealed a significant level of differentiation between islands (probably resulting from different ecological pressures coupled with drift and restricted gene flow). This is the first study on a Caribbean bird species to provide a combination of different markers for studying historical and contemporary individual flow through the archipelago. It illustrates the advantages of combining mtDNA and fast evolving neutral markers (microsatellites) with morphological data to provide a fine grain micro-evolutionary picture.

## Supporting Information

Figure S1 **Neibourgh-joining phylogenetic reconstruction of COI sequences of 11** ***Zenaida aurita*** **haplotypes.**
(DOC)

Figure S2 **Genetic structure inferred by the Bayesian clustering analysis performed with Structure.**
(DOC)

Text S1 **Ethics Statement details.**
(DOC)

Table S1 **Genetic diversity summary for 13 Zenaida Doves microsatellites for eight Caribbean islands with sample size in parenthesis.**
(DOC)

Table S2 **Haplotype definition and its spatial distribution for COI mt-DNA in** ***Zenaida aurita*** **.**
(DOC)

Table S3 **Repeatability and measurement error for tarsus length, wing chord and tail length measurements.**
(DOC)
