# Supplementary material for: Colonisation and Diversification of the Zenaida Dove (Zenaida aurita) in the Antilles: Phylogeography, Contemporary Gene Flow and Morphological Divergence
Source: PLoS One. 2013 Dec 12;8(12):e82189. doi: 10.1371/journal.pone.0082189 (PMC3861367; doi:10.1371/journal.pone.0082189)
Supplement: Figure S1 — Neibourgh-joining phylogenetic reconstruction of COI sequences of 11 Zenaida aurita haplotypes. (DOC) [file pone.0082189.s001.doc]

**Figure S1. Neibourgh-joining phylogenetic reconstruction based on K2p distance and 1000 bootstraps of COI sequences (627bp long) of 11 *Zenaida aurita* haplotypes (H_A_-H_K_).** A set of seven *Z. galapagoensis* individuals is used as an out group.
