# Supplementary material for: Colonisation and Diversification of the Zenaida Dove (Zenaida aurita) in the Antilles: Phylogeography, Contemporary Gene Flow and Morphological Divergence
Source: PLoS One. 2013 Dec 12;8(12):e82189. doi: 10.1371/journal.pone.0082189 (PMC3861367; doi:10.1371/journal.pone.0082189)
Supplement: Figure S2 — Genetic structure inferred by the Bayesian clustering analysis performed with Structure. (DOC) [file pone.0082189.s002.doc]

**Figure S2. Genetic structure inferred by the Bayesian clustering analysis performed with Structure for *K* from 2 to 8.** In each case, each colour represents a genetic cluster. Each bar corresponds to unique individual and its probability to belong in each cluster.

**
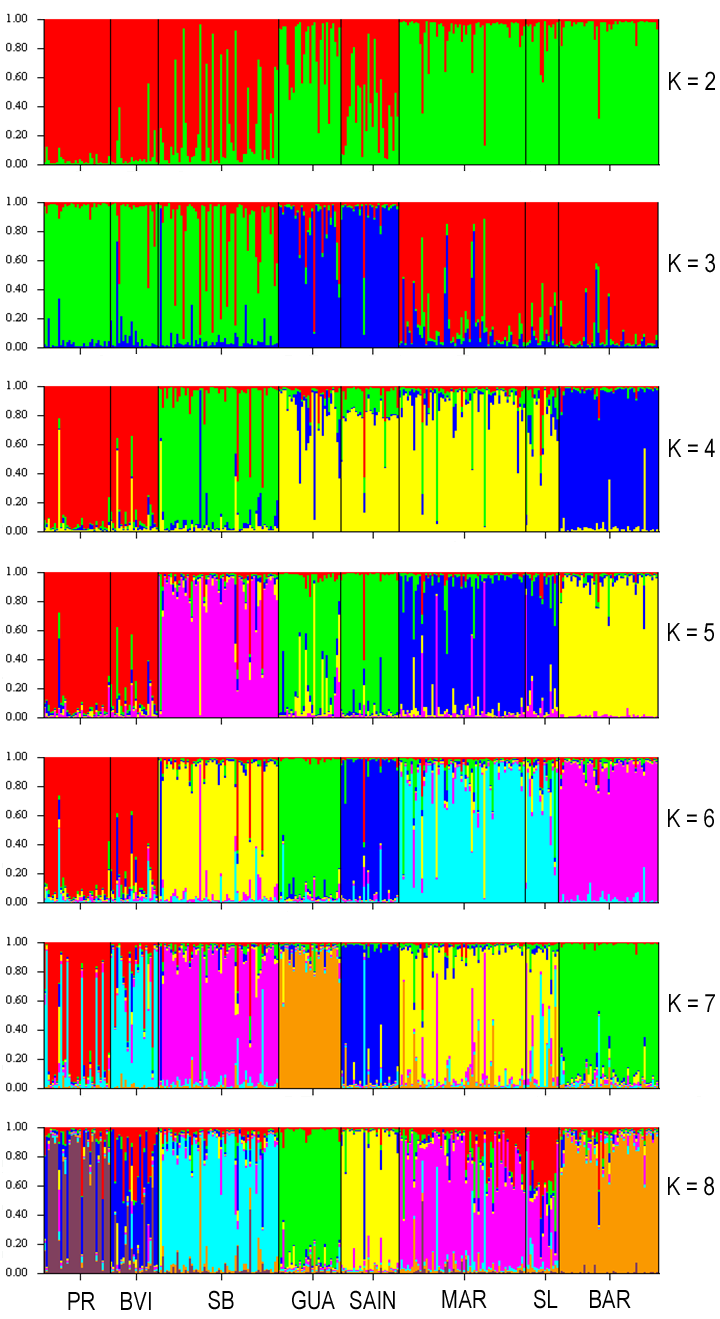
**
