# Supplementary material for: Colonisation and Diversification of the Zenaida Dove (Zenaida aurita) in the Antilles: Phylogeography, Contemporary Gene Flow and Morphological Divergence
Source: PLoS One. 2013 Dec 12;8(12):e82189. doi: 10.1371/journal.pone.0082189 (PMC3861367; doi:10.1371/journal.pone.0082189)
Supplement: Text S1 — Ethics Statement details. (DOC) [file pone.0082189.s003.doc]

**Text S1. Ethics Statement details.**

The permits were obtained from Natural Heritage Department (Ministry of Environment and Drainage) for Barbados, from the Forestry Department (Ministry of Agriculture) for Saint Lucia and from the Centre de Recherches par le Baguage des Populations d’Oiseaux (MNHN, Paris, France) for the French Antilles (Saint Barthélemy, Guadeloupe, Les Saintes, Martinique). The samples from Puerto Rico were provided by Francisco J Vilella and Huisheng Chen (MS Cooperative Fish & Wildlife Research Unit, Mississippi State University, USA), and required three permits: (1) USGS Bird Banding Laboratory Master Station (permit 22456), (2) Puerto Rico Department of Natural and Environmental Resources (permit 2010-EPE-010) and (3) Mississippi State University Institutional Animal Care and Use Committee (protocol 07-020). The samples from Guana Island (British Virgin Islands) were made available by Robert Ricklefs and Steven Latta, with support for their collection provided by Fred Sibley, James Lazell and the Falconwood Foundation. These samples only required US Fish and Wildlife Service and USDA APHIS permits for importing the samples (no specific permit was necessary in Guana Island for bird catching/blood sampling and the export of samples). Doves were mainly caught in public areas, but sometimes in private properties. In this case, permissions were obtained from the landowners to enter their property.
