# Supplementary material for: Colonisation and Diversification of the Zenaida Dove (Zenaida aurita) in the Antilles: Phylogeography, Contemporary Gene Flow and Morphological Divergence
Source: PLoS One. 2013 Dec 12;8(12):e82189. doi: 10.1371/journal.pone.0082189 (PMC3861367; doi:10.1371/journal.pone.0082189)
Supplement: Table S1 — Genetic diversity summary for 13 Zenaida Doves microsatellites for eight Caribbean islands with sample size in parenthesis. (DOC) [file pone.0082189.s004.doc]

**Table S1. Genetic diversity summary for 13 Zenaida Doves microsatellites for eight Caribbean islands with sample size in parenthesis.** Na: Number of alleles. Ar: allelic richness after rarefaction based on minimum sample size of eight individuals. *H_O_*: observed and *H_E_*: expected heterozygosities. *P*_HWE_: probability associated with Hardy Weinberg equilibrium (HWE) tests. Values in bold denote deviation from Hardy Weinberg equilibrium significant after BY’s correction.

|  | Overall (296) | PR (32) | BVI (23) | SB (58) | GUA (30) | SAIN (28) | MAR (61) | SL (16) | BAR (48) |
| --- | --- | --- | --- | --- | --- | --- | --- | --- | --- |
| ZaA4 |  |  |  |  |  |  |  |  |  |
| Na (Ar) | 17(8.55) | 10(7.39) | 8(7.01) | 11(8.03) | 7(6.24) | 7(6.31) | 11(7.56) | 6(5.59) | 6(5.69) |
| *H_O_*- *H_E_* |  | 0.67-0.68 | 0.71-0.74 | 0.72-0.79 | 0.68-0.68 | 0.67-0.81 | 0.56-0.68 | 0.62-0.73 | 0.79-0.76 |
| *P*_HWE_ |  | 0.5369 | 0.4660 | 0.0840 | 0.5768 | 0.0482 | 0.0154 | 0.2301 | 0.7492 |
| ZaA5 |  |  |  |  |  |  |  |  |  |
| Na (Ar) | 24(12.87) | 16(11.85) | 14(12.26) | 12(9.69) | 12(8.61) | 7(5.89) | 16(10.58) | 11(10.34) | 13(10.30) |
| *H_O_*- *H_E_* |  | 0.93-0.91 | 1.00-0.92 | 0.94-0.89 | 0.83-0.84 | 0.65-0.76 | 0.85-0.90 | 0.87-0.90 | 0.93-0.89 |
| *P*_HWE_ |  | 0.7773 | 1.0000 | 0.9492 | 0.5300 | 0.1256 | 0.1917 | 0.4681 | 0.1422 |
| ZaA112 |  |  |  |  |  |  |  |  |  |
| Na (Ar) | 11(6.58) | 7(5.99) | 10(8.17) | 7(6.14) | 6(5.43) | 7(6.33) | 6(5.84) | 6(5.44) | 6(5.11) |
| *H_O_*- *H_E_* |  | 0.80-0.75 | 0.82-0.85 | 0.84-0.77 | 0.83-0.81 | 0.82-0.80 | 0.79-0.82 | 0.81-0.70 | 0.95-0.75 |
| *P*_HWE_ |  | 0.8263 | 0.4463 | 0.9385 | 0.6923 | 0.6703 | 0.3816 | 0.9274 | 0.9999 |
| ZaC11 |  |  |  |  |  |  |  |  |  |
| Na (Ar) | 15(8.28) | 13(10.52) | 9(8.21) | 7(6.22) | 7(6.15) | 6(5.00) | 8(6.04) | 4(3.81) | 6(4.53) |
| *H_O_*- *H_E_* |  | 0.94-0.90 | 0.95-0.86 | 0.92-0.94 | 0.67-0.74 | 0.68-0.67 | 0.86-0.78 | 0.69-0.71 | 0.62-0.74 |
| *P*_HWE_ |  | 0.8292 | 0.9644 | 0.3917 | 0.1965 | 0.6022 | 0.9689 | 0.5329 | 0.0485 |
| ZaC12 |  |  |  |  |  |  |  |  |  |
| Na (Ar) | 21(8.95) | 13(9.87) | 10(8.80) | 9(6.49) | 8(6.51) | 8(6.46) | 5(4.64) | 6(5.96) | 11(7.50) |
| *H_O_*- *H_E_* |  | 0.52-0.88 | 0.64-0.85 | 0.67-0.77 | 0.72-0.75 | 0.81-0.64 | 0.48-0.61 | 0.69-0.78 | 0.76-0.79 |
| *P*_HWE_ |  | **< *P*** | 0.0071 | 0.0546 | 0.4389 | 0.9997 | 0.0084 | 0.2472 | 0.4021 |
| ZaD1 |  |  |  |  |  |  |  |  |  |
| Na (Ar) | 13(7.29) | 8(6.27) | 8(6.47) | 8(6.72) | 8(6.95) | 7(5.39) | 7(5.33) | 5(5.00) | 7(6.59) |
| *H_O_*- *H_E_* |  | 0.43-0.59 | 0.43-0.65 | 0.51-0.78 | 0.87-0.78 | 0.82-0.78 | 0.62-0.69 | 0.61-0.66 | 0.79-0.82 |
| *P*_HWE_ |  | 0.0087 | 0.0038 | **< *P*** | 0.9594 | 0.7717 | 0.1584 | 0.4531 | 0.3167 |
| ZaD7 |  |  |  |  |  |  |  |  |  |
| Na (Ar) | 12(7.90) | 7(5.95) | 9(7.95) | 11(7.82) | 7(6.27) | 8(7.61) | 8(7.51) | 7(6.99) | 9(7.28) |
| *H_O_*- *H_E_* |  | 0.53-0.63 | 0.74-0.85 | 0.80-0.85 | 0.72-0.78 | 0.76-0.86 | 0.68-0.83 | 0.86-0.83 | 0.79-0.80 |
| *P*_HWE_ |  | 0.1567 | 0.1341 | 0.2445 | 0.2807 | 0.108 | 0.0079 | 0.7544 | 0.5631 |
| ZaD11 |  |  |  |  |  |  |  |  |  |
| Na (Ar) | 11(6.39) | 10(7.73) | 7(6.05) | 7(5.73) | 5(4.32) | 3(3.00) | 7(5.49) | 4(4.00) | 8(6.21) |
| *H_O_*- *H_E_* |  | 0.61-0.78 | 0.78-0.71 | 0.63-0.76 | 0.68-0.69 | 0.72-0.67 | 0.72-0.75 | 0.36-0.73 | 0.80-0.80 |
| *P*_HWE_ |  | 0.0074 | 0.8802 | 0.0142 | 0.5125 | 0.778 | 0.3163 | 0.0032 | 0.5678 |
| ZaD104 |  |  |  |  |  |  |  |  |  |
| Na (Ar) | 12(7.83) | 7(6.06) | 9(7.95) | 8(7.14) | 7(6.88) | 6(4.39) | 7(5.42) | 6(6.00) | 9(7.30) |
| *H_O_*- *H_E_* |  | 0.59-0.77 | 0.65-0.86 | 0.71-0.80 | 0.70-0.85 | 0.64-0.66 | 0.74-0.79 | 0.79-0.84 | 0.80-0.84 |
| *P*_HWE_ |  | 0.0123 | 0.0086 | 0.0334 | 0.027 | 0.4803 | 0.2119 | 0.399 | 0.2751 |
| ZaD105 |  |  |  |  |  |  |  |  |  |
| Na (Ar) | 9(6.29) | 9(7.16) | 7(6.30) | 6(5.86) | 6(5.33) | 4(3.39) | 6(5.23) | 6(6.00) | 6(4.71) |
| *H_O_*- *H_E_* |  | 0.72-0.81 | 0.78-0.78 | 0.72-0.80 | 0.73-0.75 | 0.61-0.59 | 0.71-0.74 | 0.92-0.82 | 0.61-0.60 |
| *P*_HWE_ |  | 0.107 | 0.5907 | 0.0791 | 0.4838 | 0.6584 | 0.3212 | 0.9199 | 0.6612 |
| ZaD108 |  |  |  |  |  |  |  |  |  |
| Na (Ar) | 14(9.15) | 11(10.13) | 9(8.51) | 9(7.21) | 10(8.27) | 7(6.26) | 8(6.19) | 5(4.81) | 10(8.87) |
| *H_O_*- *H_E_* |  | 0.58-0.91 | 0.38-0.88 | 0.59-0.81 | 0.72-0.85 | 0.67-0.75 | 0.72-0.82 | 0.56-0.71 | 0.85-0.89 |
| *P*_HWE_ |  | **< *P*** | **< *P*** | **< *P*** | 0.0543 | 0.1973 | 0.0329 | 0.1267 | 0.3017 |
| ZaD119 |  |  |  |  |  |  |  |  |  |
| Na (Ar) | 14(7.64) | 9(7.03) | 9(7.50) | 8(6.78) | 9(6.74) | 9(8.11) | 7(6.43) | 6(5.86) | 10(6.89) |
| *H_O_*- *H_E_* |  | 0.61-0.81 | 0.69-0.84 | 0.82-0.83 | 0.69-0.79 | 0.67-0.84 | 0.72-0.82 | 0.93-0.79 | 0.85-0.76 |
| *P*_HWE_ |  | 0.004 | 0.0502 | 0.5130 | 0.1429 | 0.0108 | 0.0302 | 0.9778 | 0.9702 |
| ZaD121 |  |  |  |  |  |  |  |  |  |
| Na (Ar) | 15(8.82) | 11(9.39) | 10(9.00) | 8(6.45) | 7(5.81) | 8(7.27) | 13(8.32) | 9(8.70) | 10(9.10) |
| *H_O_*- *H_E_* |  | 0.65-0.89 | 0.56-0.89 | 0.65-0.81 | 0.80-0.80 | 0.92-0.86 | 0.78-0.85 | 0.80-0.88 | 0.73-0.89 |
| *P*_HWE_ |  | **< *P*** | **< *P*** | 0.0021 | 0.5947 | 0.9186 | 0.0933 | 0.2606 | 0.0032 |
| All (mean values) |  |  |  |  |  |  |  |  |  |
| Na (Ar) |  | 10.08 | 9.15 | 8.54 | 7.61 | 6.69 | 8.38 | 6.23 | 8.54 |
| *H_O_*- *H_E_* |  | 0.66-0.79 | 0.70-0.82 | 0.73-0.81 | 0.74-0.78 | 0.73-0.74 | 0.71-0.77 | 0.73-0.77 | 0.79-0.79 |
| *P*_HWE_ |  | **< *P*** | **< *P*** | **< *P*** | 0.0357 | 0.1535 | **< *P*** | 0.0628 | 0.2519 |
