# Supplementary material for: Colonisation and Diversification of the Zenaida Dove (Zenaida aurita) in the Antilles: Phylogeography, Contemporary Gene Flow and Morphological Divergence
Source: PLoS One. 2013 Dec 12;8(12):e82189. doi: 10.1371/journal.pone.0082189 (PMC3861367; doi:10.1371/journal.pone.0082189)
Supplement: Table S2 — Haplotype definition and its spatial distribution for COI mt-DNA in Zenaida aurita . (DOC) [file pone.0082189.s005.doc]

**Table S2. Haplotype definition and its spatial distribution for COI mt-DNA (627 pb) in *Zenaida aurita*.** GenBank: Accession JN639022-32. Position: the number represents the position of polymorphic sites relative Genbank sequence Accession number JN639022 (denotes homology to reference haplotype H_A_). Island: sample size for each haplotype for each sampled island. N: total sample size for each haplotype.

| Haplotype |  | Position | |  |  |  |  |  |  |  |  |  | Island | |  |  |  |  |  |  | N |
| --- | --- | --- | --- | --- | --- | --- | --- | --- | --- | --- | --- | --- | --- | --- | --- | --- | --- | --- | --- | --- | --- |
|  |  | 16 | 220 | 229 | 232 | 277 | 350 | 372 | 436 | 479 | 547 |  | PR | BVI | SB | GUA | SAIN | MAR | SL | BAR |
| H_A_ |  | T | C | G | T | C | G | C | A | C | A |  | 2 | 4 | 52 | 24 | 27 | 41 | 13 | 31 | 194 |
| H_B_ |  | C | - | - | - | - | - | - | - | - | - |  | - | - | - | - | - | 3 | - | - | 3 |
| H_C_ |  | - | T | - | - | - | - | - | - | - | - |  | - | - | - | - | - | - | - | 16 | 16 |
| H_D_ |  | - | - | - | - | T | - | - | - | - | - |  | - | - | - | - | - | - | - | 1 | 1 |
| H_E_ |  | - | - | - | - | - | - | T | - | - | - |  | - | - | - | - | - | 1 | - | - | 1 |
| H_F_ |  | - | - | A | - | - | - | - | - | - | T |  | 7 | 5 | - | - | - | - | - | - | 12 |
| H_G_ |  | - | - | A | C | - | - | - | - | - | T |  | 9 | 14 | - | - | - | - | - | - | 23 |
| H_H_ |  | - | - | A | - | - | A | - | C | - | - |  | 1 | - | - | - | - | - | - | - | 1 |
| H_I_ |  | - | - | A | - | - | - | - | C | - | T |  | 1 | - | - | - | - | - | - | - | 1 |
| H_J_ |  | - | - | A | C | - | - | - | C | - | T |  | 1 | - | - | - | - | - | - | - | 1 |
| H_K_ |  | - | - | A | C | - | A | - | - | G | T |  | 1 | - | - | - | - | - | - | - | 1 |
