# Supplementary material for: Colonisation and Diversification of the Zenaida Dove (Zenaida aurita) in the Antilles: Phylogeography, Contemporary Gene Flow and Morphological Divergence
Source: PLoS One. 2013 Dec 12;8(12):e82189. doi: 10.1371/journal.pone.0082189 (PMC3861367; doi:10.1371/journal.pone.0082189)
Supplement: Table S3 — Repeatability and measurement error for tarsus length, wing chord and tail length measurements. (DOC) [file pone.0082189.s006.doc]

**Table S3. Repeatability (R) for tarsus length, wing chord and tail length measurements with 95% confidence intervals (95% CI) and statistical significance of likelihood ratio tests (*P*), per islands and overall (N = 235).** Measurement error (ME in %) defined as (1 – R) x 100 is also presented.

|  | Tarsus length | | | | Wing chord | | | | Tail length | | | |
| --- | --- | --- | --- | --- | --- | --- | --- | --- | --- | --- | --- | --- |
| Island | R | 95% CI | *P* | ME | R | 95% CI | *P* | ME | R | 95% CI | *P* | ME |
| SB | 0.950 | 0.918-0.970 | < 0.0001 | 5.00 | 0.962 | 0.936-0.977 | < 0.0001 | 3.80 | 0.937 | 0.901-0.961 | < 0.0001 | 6.30 |
| GUA | 0.988 | 0.974-0.995 | < 0.0001 | 1.20 | 0.983 | 0.964-0.991 | < 0.0001 | 1.70 | 0.977 | 0.950-0.989 | < 0.0001 | 2.30 |
| SAIN | 0.988 | 0.975-0.994 | < 0.0001 | 1.20 | 0.983 | 0.967-0.992 | < 0.0001 | 1.70 | 0.982 | 0.961-0.991 | < 0.0001 | 1.80 |
| MAR | 0.965 | 0.941-0.980 | < 0.0001 | 3.50 | 0.941 | 0.900-0.967 | < 0.0001 | 5.90 | 0.950 | 0.915-0.970 | < 0.0001 | 5.00 |
| SL | 0.990 | 0.97-0.996 | < 0.0001 | 1.00 | 0.980 | 0.936-0.993 | < 0.0001 | 2.00 | 0.955 | 0.868-0.983 | < 0.0001 | 4.50 |
| BAR | 0.953 | 0.919-0.974 | < 0.0001 | 4.70 | 0.953 | 0.920-0.972 | < 0.0001 | 4.70 | 0.935 | 0.884-0.961 | < 0.0001 | 6.50 |
| Overall | 0.970 | 0.962-0.977 | < 0.0001 | 3.00 | 0.972 | 0.965-0.978 | < 0.0001 | 2.80 | 0.978 | 0.971-0.983 | < 0.0001 | 2.20 |
